# The *M/V X-Press Pearl* Nurdle Spill: Contamination of Burnt Plastic and Unburnt Nurdles along Sri Lanka’s Beaches

**DOI:** 10.1021/acsenvironau.1c00031

**Published:** 2021-11-29

**Authors:** Asha de Vos, Lihini Aluwihare, Sarah Youngs, Michelle H. DiBenedetto, Collin P. Ward, Anna P. M. Michel, Beckett C. Colson, Michael G. Mazzotta, Anna N. Walsh, Robert K. Nelson, Christopher M. Reddy, Bryan D. James

**Affiliations:** † Oceanswell, 9 Park Gardens, Colombo 5 00500, Sri Lanka; ‡ The Oceans Institute, University of Western Australia, 35 Stirling Highway, Perth, WA 6009, Australia; § Scripps Institution of Oceanography, University of California San Diego, La Jolla, California 92093, United States; ∥ Department of Applied Ocean Physics and Engineering, Woods Hole Oceanographic Institution, Woods Hole, Massachusetts 02543, United States; ⊥ Department of Mechanical Engineering, University of Washington, Seattle, Washington 98195, United States; # Department of Marine Chemistry and Geochemistry, Woods Hole Oceanographic Institution, Woods Hole, Massachusetts 02543, United States; ¶ Department of Mechanical Engineering, Massachusetts Institute of Technology, Cambridge, Massachusetts 02139, United States; △ MIT−WHOI Joint Program in Oceanography/Applied Ocean Science & Engineering, Cambridge and Woods Hole, Massachusetts 02139, United States; ▼ Department of Civil and Environmental Engineering, Massachusetts Institute of Technology, Cambridge, Massachusetts 02139, United States

**Keywords:** microplastic, pyroplastic, pollution, ship fire, contaminants, oil, maritime accident, citizen science

## Abstract

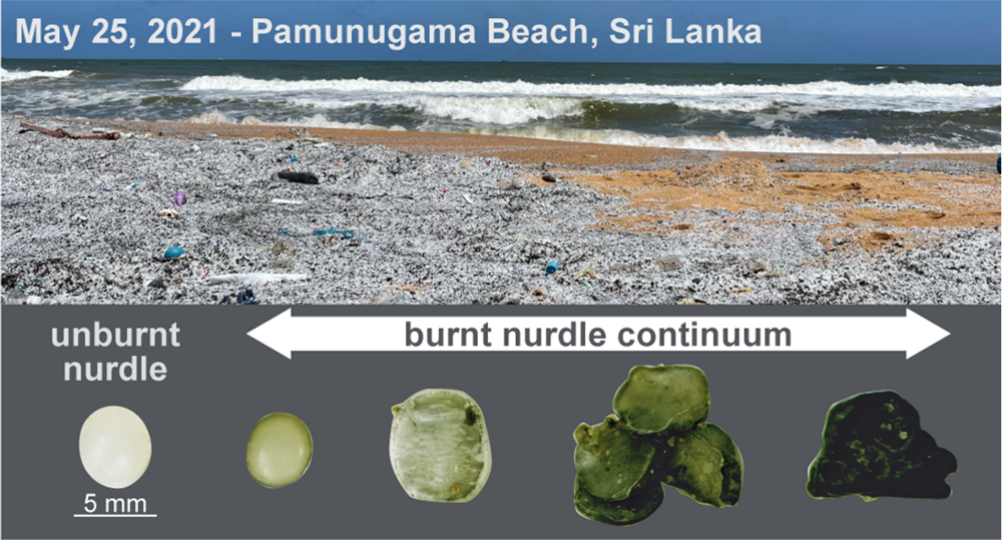

In May 2021, the *M/V X-Press Pearl* cargo ship caught fire 18 km off the west coast of Sri Lanka and spilled ∼1680 tons of spherical pieces of plastic or “nurdles” (∼5 mm; white in color). Nurdles are the preproduction plastic used to manufacture a wide range of end products. Exposure to combustion, heat, and chemicals led to agglomeration, fragmentation, charring, and chemical modification of the plastic, creating an unprecedented complex spill of visibly burnt plastic and unburnt nurdles. These pieces span a continuum of colors, shapes, sizes, and densities with high variability that could impact cleanup efforts, alter transport in the ocean, and potentially affect wildlife. Visibly burnt plastic was 3-fold more chemically complex than visibly unburnt nurdles. This added chemical complexity included combustion-derived polycyclic aromatic hydrocarbons. A portion of the burnt material contained petroleum-derived biomarkers, indicating that it encountered some fossil-fuel products during the spill. The findings of this research highlight the added complexity caused by the fire and subsequent burning of plastic for cleanup operations, monitoring, and damage assessment and provides recommendations to further understand and combat the impacts of this and future spills.

On May 20, 2021, a fire broke out on the deck of the *M/V X-Press Pearl* cargo ship, while anchored 18 km off the west coast of Sri Lanka and waiting to offload a container leaking nitric acid (Figure 1). The ship manifest showed it carried 1486 containers, 1214 of which were loaded with an assortment of raw materials, hazardous chemicals, and finished products (Supporting Information).^1^ The following day, an explosion exacerbated an already complex, challenging, and domino-effect-driven crisis for the ship’s crew and subsequent responders.^2^ The crew and responders were tasked with fighting the fire, containing the nitric acid leak, evacuating from the ship, keeping the ship afloat, and managing spills of cargo and fuel. Moreover, this occurred during the COVID-19 pandemic, with movement restricted by curfew laws and the seasonal southwest monsoon. While the fire was doused by June 1, salvage operations were insufficient to prevent the ship from wholly sinking by June 17 (Figure 1).^3^ Throughout the crisis, containers burned, fell overboard with some sinking, and others stranding on beaches and spilling their contents in the process.^4^

**Figure 1:**
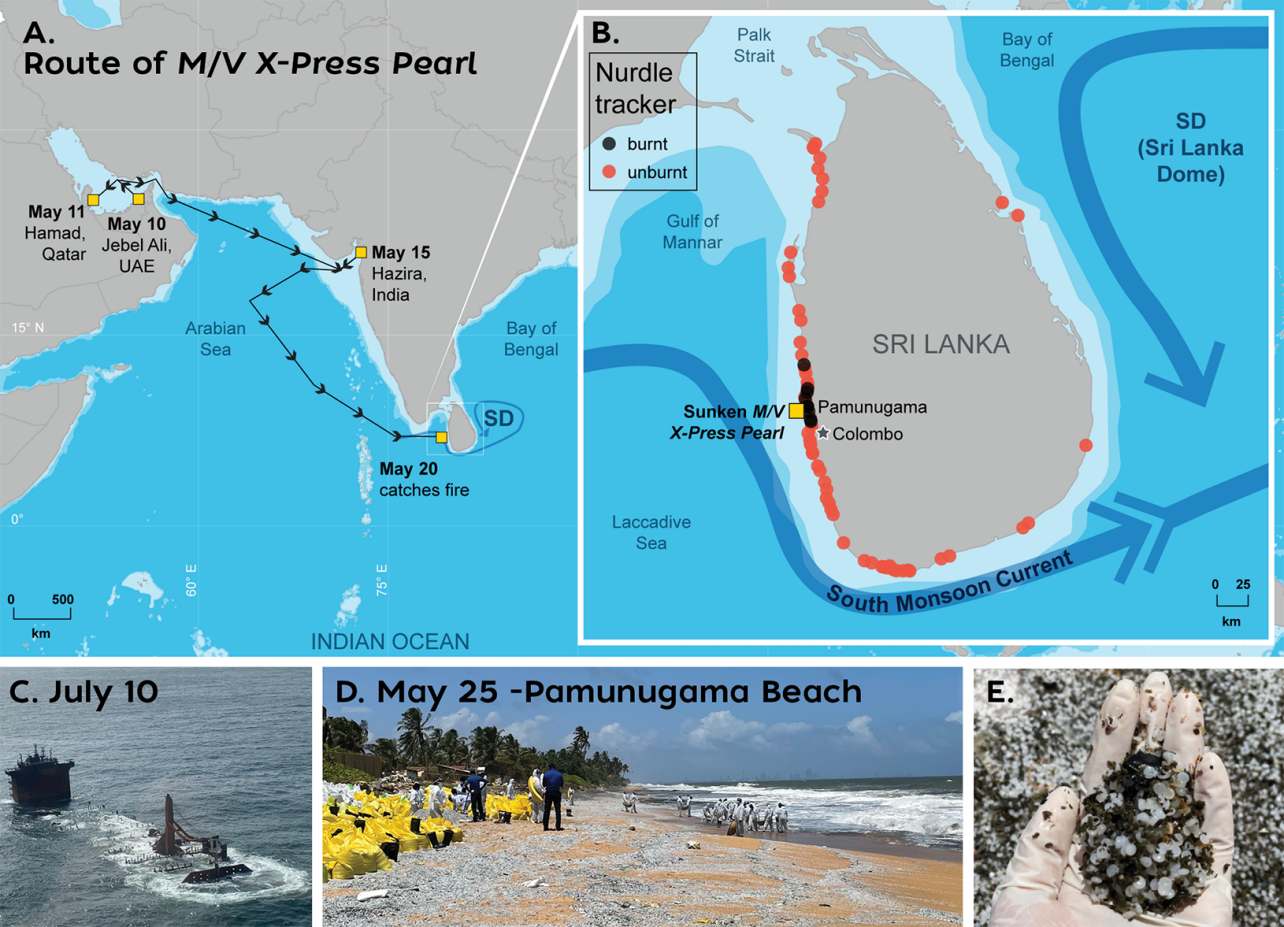
Travel route^7^ of the *M/V X-Press Pearl* leading up to the spill (A) and map of crowd-sourced nurdle sightings collated by Oceanswell^5^ between May 29 to July 11, 2021 (B). Photographs of (C) the ship after sinking taken on July 10 (photo credit: Conor Bolas, ITOPF), of (D) burnt plastic and unburnt nurdles that washed ashore onto Pamunugama beach, and of (E) a handful of them both; photographs were taken on May 25. Full-sized photographs are available in Figures S1 and S2.

Within 5 days of the fire, white opaque nurdles (∼5 mm) as well as irregularly shaped dark plastic pieces, both smaller (at least 0.5 mm) and larger (at least 6 cm) than the nurdles that spilled from the ship, reached the Sri Lankan shore (Figures S1–S4). Nurdles are the preproduction plastic used to manufacture a wide range of end products. The altered pieces of plastic were attributed to the ship fire melting and burning a portion of the nurdles and possibly other plastic cargo. Following the spill, crowd-sourced data collated by Oceanswell^5^ tracked and observed nurdles reaching as far as the east coast of Sri Lanka by early July (Figure 1). The crowd-sourced data were filtered and verified using photographs and results of a particle tracking model of the spill.^6,7^ Based on the crowd-sourced data, by July 11, the burnt plastic was only observed over a ∼50 km stretch of the west coast close to the ship (Figure 1). This surprising finding raises several questions about the events of the spill and the processes that restricted their dispersion. While container-related nurdle spills have occurred in the past,^8−10^ the burnt plastic presents an unknown challenge for the cleanup, monitoring, and damage assessment of this spill, thereby demanding more in-depth analysis of and consideration for the material that spilled. As highlighted in the United Nations Environment Programme (UNEP) environmental assessment report,^4^ this work looks to (i) characterize how the ship fire changed the physical and chemical properties of the nurdles washing ashore, (ii) understand how the burnt plastic could differentiate this spill from previous nurdle spills, and (iii) provide recommendations for current and future responses. To meet these goals, we analyzed plastic samples collected on May 25, 2021 from Pamunugama Beach, Sri Lanka (Figure 1).

## Fire-Induced Changes in the Nurdles Created a Continuum of Plastic Pollution

### Appearance

**Figure 2:**
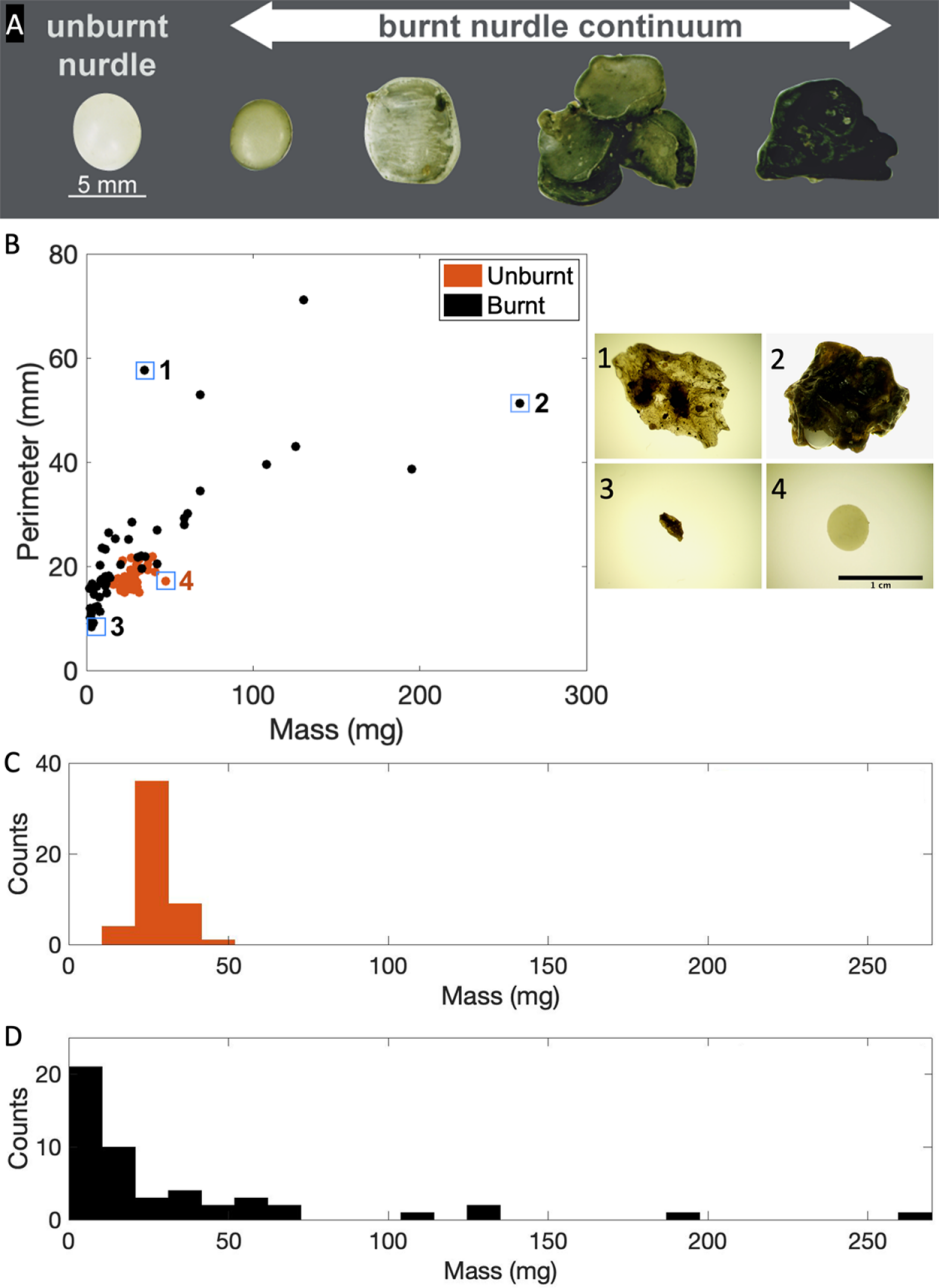
Recovered plastic spanned a continuum from white unburnt nurdles to black burnt plastic (A). Comparison of the projected perimeter and mass for a sampling of the burnt plastic and unburnt nurdles (B). Histograms of the mass distribution for a sampling of unburnt nurdles (C) and of burnt plastic (D). For these analyses, degraded nurdles and burnt plastic were combined because this analysis intended to show the variability of the plastic along the burnt nurdle continuum. These measurements should not be considered exhaustive; for instance, the plastic that was exposed to fire can be larger than represented here; see Figure S2.

Visual inspection showed the plastic varied in color, shape, and size largely due to initial exposure to the fire. Principally, the plastic could be coarsely separated into three groups (Figure 2A): (i) “unburnt” nurdles that were white and opaque; (ii) “degraded” nurdles that were yellowed, darkened, and speckled with black inclusions much like nurdles that have been weathered;^11,12^ and (iii) “burnt” plastic pieces that were completely black or dark green. As with color, the fire created a continuum of shapes and sizes. The unburnt nurdles were generally homogeneous, spherical, and smooth with some bearing divots. In contrast, the burnt plastic had characteristics of pyroplastic,^13^ which included increased heterogeneity, angulation, roughness, agglomeration, indentation, and brittleness. Degraded nurdles reside between these two extremes. The qualitative differences between the visibly unburnt nurdles and those along the burnt nurdle continuum (Figure 2A) were confirmed by image-based quantification of several morphometrics that were measured for a subset of 50 unburnt nurdles and 50 burnt plastic pieces (Figures 2B–D and S5). The continuum of shapes and sizes is likely due to both agglomeration and fragmentation: the result of heating, burning, mechanical breakdown, or other extreme processes that acted on the plastic.

### Density and Buoyancy

Unlike the broad range of possible appearances, the plastic had one of two positively buoyant densities, either ∼0.93 or ∼0.96 g/mL. Low-density polyethylene (LDPE) and high-density polyethylene (HDPE) have densities from 0.91 to 0.94 and 0.94 to 0.97 g/mL, respectively,^14,15^ suggesting the plastic washing ashore was polyethylene (PE). This is corroborated by the cargo manifest (Supporting Information), analysis by attenuated total reflection Fourier transform infrared spectroscopy of an unburnt nurdle^16^ (Figure S6), and findings from the July UNEP report.^4^

Transport and fate of plastic in the ocean is a function of the plastic’s buoyancy, which dictates how easily the plastic can be mixed below the surface of the ocean.^17^ The effective buoyant forces for the plastic analyzed in Figure 2 were calculated, which indicated a narrow distribution for the unburnt nurdles due to their consistent properties, whereas the burnt plastic showed a larger distribution of values (Figure S7). The most buoyant particles were all larger and burnt.

### Inorganic and Solvent-Extractable Material

No measurable material remained after heating at 450 °C for 4 h, indicating that the content of the unburnt nurdles was organic, combustible material.^18^ In contrast, excised material from large agglomerations (>10× larger than nurdles) of burnt plastic (Figure S2), referred to as a combustion remnant, contained 1.2 ± 0.2% (*n* = 3) noncombusted materials composed of “char-like” pieces and fine ash. The percentage and color of dichloromethane-extractable material from a fixed mass of nurdles increased for burnt plastic compared to that of unburnt nurdles. Conversely, there was only a slight difference in extractable mass between the unburnt and degraded nurdles. The extractable mass ranged from <0.1 to 3.1% for visually unburnt nurdles and burnt plastic, respectively (*n* = 2 for each group along the continuum). The combustion remnants had 3.0–5.0% extractable material (*n* = 2). The colors of the extracts largely corresponded to the extractable masses (Figure S8). Bulk elemental analysis for carbon, hydrogen, and nitrogen (Figure S9) mirrored the aforementioned results. The hydrogen-to-carbon (H/C) ratio was 1.94 for the unburnt nurdles, approaching the theoretical value of 2.01 for pure PE and increased along the burnt nurdle continuum to 2.14 for the combustion remnant. This increase could be due to thermal degradation, leading to shorter carbon chains with higher H/C ratios or from the production of hydroxyl groups.

**Figure 3:**
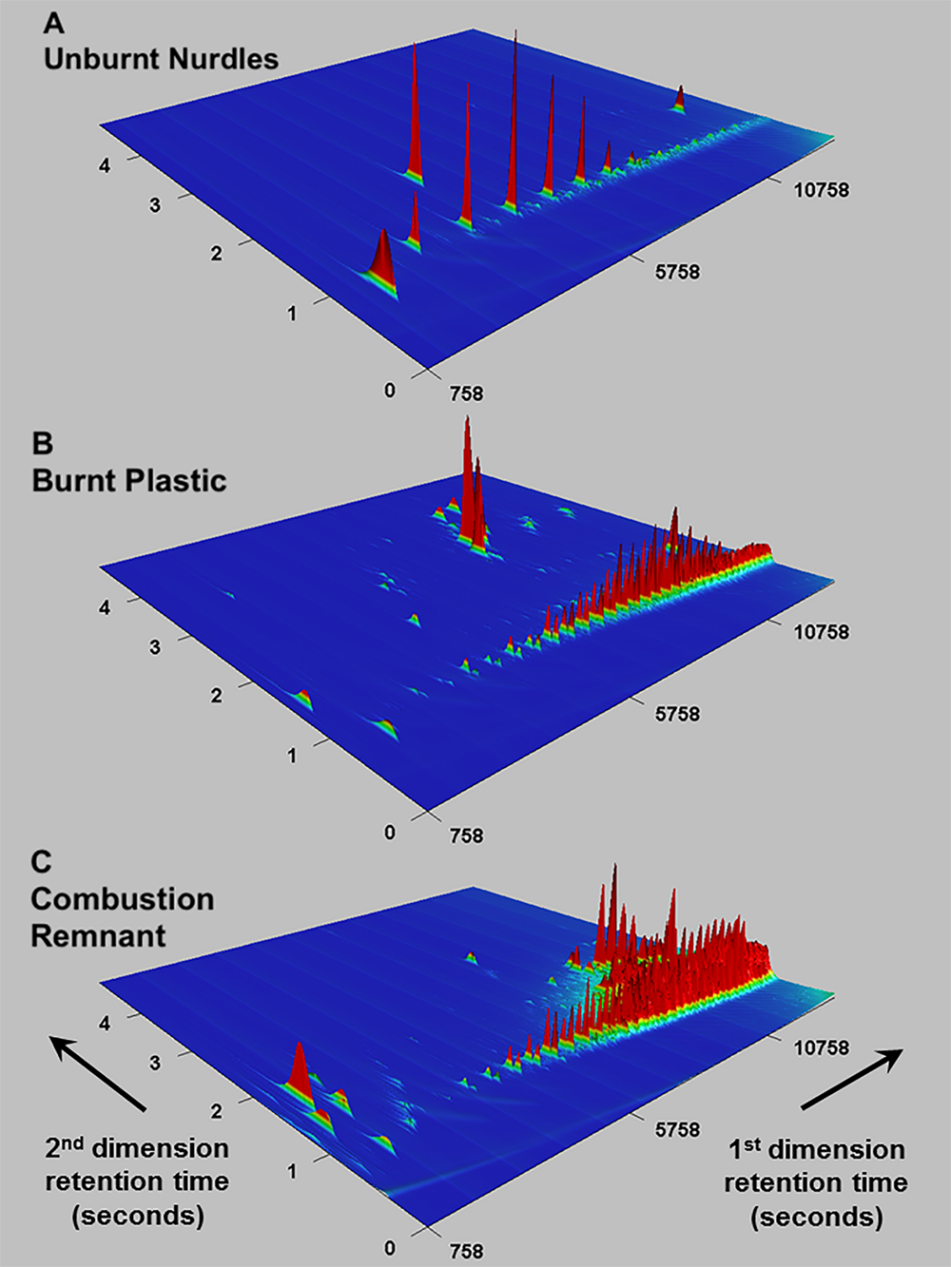
GC×GC-FID chromatograms of dichloromethane-extractable material from unburnt nurdles, 400 peaks (A), burnt plastic, 1000 peaks (B), and combustion remnant, 1300 peaks (C). Each chromatogram is scaled to itself. Details of the features are annotated in the Supporting Information.

Comprehensive gas chromatography (GC×GC) analysis of the dichloromethane extracts revealed that combustion altered the complexity and distribution of compound classes. Compared to the visibly unburnt nurdle samples, chemical complexity, as defined by the number of detected peaks, increased by approximately 2- and 3-fold in the visibly burnt plastic and combustion remnant samples, respectively (Figure 3). The unburnt nurdles were dominated by even-numbered (with trace amounts of odd-numbered) alkenes from 10 to 26 carbon atoms. Accompanying each alkene were low levels of *n*-alkanes (Figures 3A and S10A). We had expected a greater amount of *n-*alkanes than alkenes; nevertheless, this signature matches that of solvent-extracted PE^19−21^ and is not observed in solvent extracts of other plastics listed on the ship manifest (e.g., PP,^19^ PVC,^19^ PS,^22^ PA,^23^ PET^24^). However, we cannot definitively rule out the possibility that the visibly unburnt nurdles were exposed to elevated temperatures, leading to preferential enrichments of alkenes and alkadienes.^23,25^ The GC×GC chromatograms of the burnt plastic (Figures 3B and S10B) and combustion remnant (Figures 3C and S10C) contained “fairways” of alkadiene, alkene, and alkane triplets (Figure S11) indicative of partial combustion at more elevated temperatures.^23^ Oxygen-containing analogues (e.g., aldehydes, ketones) were not observed. Another indicator for partial combustion was the presence of parent polycyclic aromatic hydrocarbons (PAHs), naphthalene, phenanthrene, fluoranthene, and pyrene (Figure S12).^26,27^ All samples analyzed by GC×GC included 2,4-di-*tert*-butylphenol (tentatively identified by placement on the two-dimensional gas chromatogram, mass spectrum, and accurate mass of fragments). This compound is known to be a breakdown product of the PE processing stabilizer, [tris(2,4-di-*tert*-butylphenyl)phosphite] (Irgafos 168).^28,29^ Transformation products of phenolic antioxidants are largely responsible for the yellowing of polyethylene.^30^ No phthalates were detected. While no petroleum-derived alkylated PAHs were detected, the combustion remnant contained fossil-fuel biomarkers (Figure S13) (e.g., hopanes and steranes),^31^ suggesting that this plastic encountered the ship’s underway fuel or lubricating oils before reaching the shoreline. This assessment was not meant to be comprehensive, and much more remains to be done; however, these preliminary findings can provide valuable forensic evidence and insight into the accident to inform efforts while the response is ongoing.

The continuum of chemical and physical changes of the nurdles that was identified in this study paves the way for more quantitative metrics to describe how the preproduction plastic was affected by the combined effects of the ship fire and chemicals released from the *X-Press Pearl*.

## Presence of Burnt Plastic Complicates the Spill

**Figure 4:**
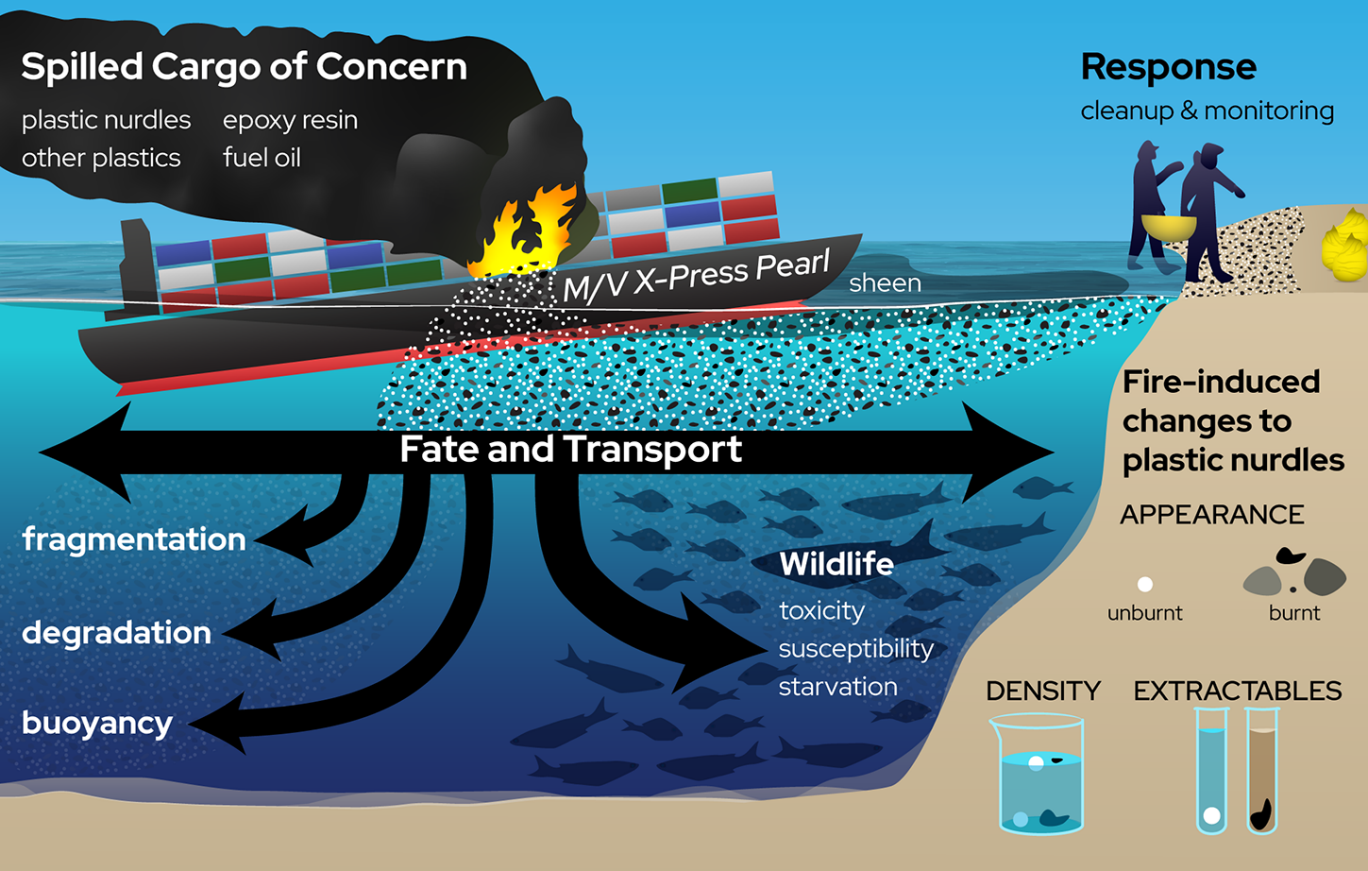
Concerns and potential impacts following the *M/V X-Press Pearl* nurdle spill.

Chronic “nurdling” of coastlines is part of an ongoing global trend of plastic entering the ocean and has been recognized as a potential problem since the 1970s when oceanographers first identified nurdles in coastal waters.^32−37^ Unlike past nurdle spills though, this spill released burnt plastic, a type of litter that has only recently been documented in the ocean,^13^ posing uncertainties for (i) the response activities, (ii) the fate and transport of the plastic, and (iii) the potential impact on wildlife (Figure 4).

### Impacts of Fire on Rescue Activities

**Figure 5:**
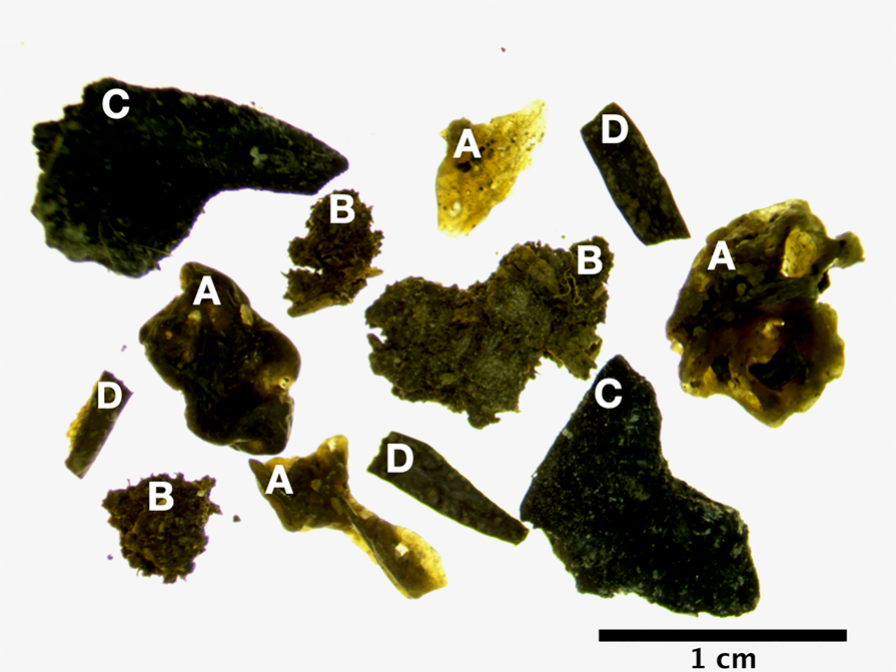
Burnt plastic (A) looks similar to natural materials common to beaches such as soil (B), wood (C), and seagrass (D).

The fire changed the nurdles, leading to a wide range of appearances that complicated already challenging cleanup efforts, monitoring, and damage assessment for nurdle spills.^4,10,38,39^ On the beach, unburnt nurdles have been reported to be easy to identify;^40^ though, unburnt nurdles may blend in with shells and sand (Figure S4). Similarly, the burnt plastic may camouflage among natural materials (Figure 5). The limited distribution of burnt plastic found on the beaches (Figure 1) could be due to the material evading detection. However, citizen scientists informed about the burnt plastic pieces have not seen them outside the isolated section of coastline, suggesting another explanation could be responsible for the segregation of the burnt plastic.

Alternatively, the distributions of burnt plastic and unburnt nurdles along the Sri Lankan coastline support the possibility that differential transport in the ocean is affected by the physical alterations caused by the fire (Figures 2 and S5 and S7). It is reasonable to conclude that the increased heat from the fire melted and agglomerated the nurdles. These large nurdle agglomerations are more buoyant than the smaller unburnt nurdles and will therefore be less readily mixed below the surface of the ocean. Considering that coastal ocean currents are vertically sheared, small changes to a particle’s vertical position in the water column can lead to large changes in horizontal transport.^41^ Because on-shore wave-driven transport and windage are strongest at the surface of the ocean, the most buoyant particles would likely have been transported on-shore the fastest, limiting the time available for their dispersal, consistent with the burnt plastic being found in a confined region (Figure 1B). Once on-shore, swash-zone processes (e.g., wave breaking) may have contributed to increased fragmentation of the more brittle burnt plastic,^42^ explaining the presence of both small and large burnt plastic pieces on the beach.

Beyond buoyancy, the shape differences between the burnt plastic and unburnt nurdles is likely important, as nonsphericity can control the transport of particles in the ocean.^43^ The observed differential fate of the burnt plastic and unburnt nurdles highlights that particle size and shape can be a driving factor in determining transport. However, the exact pathways of this distinction are unknown, highlighting the need for detailed field measurements and controlled laboratory and numerical experiments.

### Exposure of Plastics to Wildlife

Increasingly, aquatic organisms have been documented to actively and passively interact with and ingest plastic.^44−52^ Because of its variable appearance, the burnt plastic may resemble different types of prey and be ingested and translocated, differently affecting how wildlife interact with the plastic.^53,54^ Adding to this, the quantity of smaller burnt pieces could increase due to the plastic’s brittleness, creating a fraction of plastic of reduced size that could be more harmful in some cases or egested more efficiently in other cases.^55,56^ Moreover, during the fire, the plastic was exposed to carcinogenic combustion products and developed additive degradation products^57^ as revealed by GC×GC analysis, potentiating the burnt plastic as a vector with unknown bioavailability for carcinogenic PAHs and other pollutants.^58−67^ Collectively, these features differentiate this spill from past nurdle spills and warrant investigation of how the toxicity varies along the burnt nurdle continuum.

## Recommendations for an Unprecedented Nurdle Spill

As of July 14, ∼53,000 bags of unburnt nurdles, burnt plastic, and other debris have been cleaned from Sri Lankan beaches. Despite these efforts, there is concern that this incident may have affected some marine species, although results to verify this have yet to be released by the relevant authorities. Adding to this uncertainty, there has yet to be a reported accounting of the ship’s cargo.^4^ With the earliest estimates being late 2021 for removal of the wreckage, there is significant potential for further impacts.^4,68^ Here, we provide five key recommendations to aid in ongoing and future responses to and damage assessment of this unprecedented nurdle spill that can also inform responses to future spills.

(1) Educate and empower citizen scientists to continue safely reporting sightings of nurdles to inform cleanup efforts.
(2) Account for the cargo initially onboard the vessel to better gauge long-term threats from the wreck.
(3) Continue urgency for clean-ups after a spill, revise response efforts to account for any variability of spilled nurdles, and minimize removal of natural organic matter from the beaches.
(4) Encourage locally led research efforts on nurdle samples to assess their physical and chemical properties, fate, and transport in the ocean and toxicity to aquatic life.
(5) Prepare a robust baseline of plastic pollution stemming from the spill across multiple field sites and time points and compare it to recent prespill reports of microplastic pollution.^40,69−73^

## Supporting Information Available

Photographs from the spill, characterization of nurdle morphometrics, ATR-FTIR spectra of an unburnt nurdle, estimated buoyancy of nurdles, bulk elemental analysis of the plastic, photographs of solvent extracts, annotated GC×GC chromatograms, materials and methods (PDF)
Ship manifest (XLSX)
