# Supplementary material for: The M/V X-Press Pearl Nurdle Spill: Contamination of Burnt Plastic and Unburnt Nurdles along Sri Lanka’s Beaches
Source: ACS Environ Au. 2021 Nov 29;2(2):128–35. doi: 10.1021/acsenvironau.1c00031 (PMC10114858; doi:10.1021/acsenvironau.1c00031)
Supplement: Supplementary file 1 — vg1c00031_si_001.pdf [file vg1c00031_si_001.pdf]

Supporting Information

The *M/V X-Press Pearl* nurdle spill: Contamination of burnt plastic and unburnt nurdles along Sri Lanka's beaches

Asha de Vos[a, b, *], Lihini Aluwihare[c], Sarah Youngs[d], Michelle H. DiBenedetto[e], Collin P. Ward[f], Anna P. M. Michel[d, *], Beckett C. Colson[d, g, h], Michael G. Mazzotta[f], Anna N. Walsh[f, h, i], Robert K. Nelson[f], Christopher M. Reddy[f], Bryan D. James[f]

[a] Oceanswell, 9 Park Gardens, Colombo 5, Sri Lanka
[b] The Oceans Institute, University of Western Australia, 35 Stirling Highway, Perth, WA, 6009, Australia
[c] Scripps Institution of Oceanography, University of California San Diego, La Jolla, California, 92093, United States
[d] Department of Applied Ocean Physics and Engineering, Woods Hole Oceanographic Institution, Woods Hole, Massachusetts 02543, United States
[e] Department of Mechanical Engineering, University of Washington, Seattle, Washington 98195, United States
[f] Department of Marine Chemistry and Geochemistry, Woods Hole Oceanographic Institution, Woods Hole, Massachusetts 02543, United States
[g] Department of Mechanical Engineering, Massachusetts Institute of Technology, Cambridge, Massachusetts 02139, United States
[h] MIT-WHOI Joint Program in Oceanography/Applied Ocean Science & Engineering, Cambridge and Woods Hole, Massachusetts, United States
[i] Department of Civil and Environmental Engineering, Massachusetts Institute of Technology, Cambridge, Massachusetts 02139, United States

*Corresponding author information:
Asha de Vos: Email: asha@oceanswell.org
Anna P. M. Michel: Email: amichel@whoi.edu

No. of pages: 16
No. of figures: 13

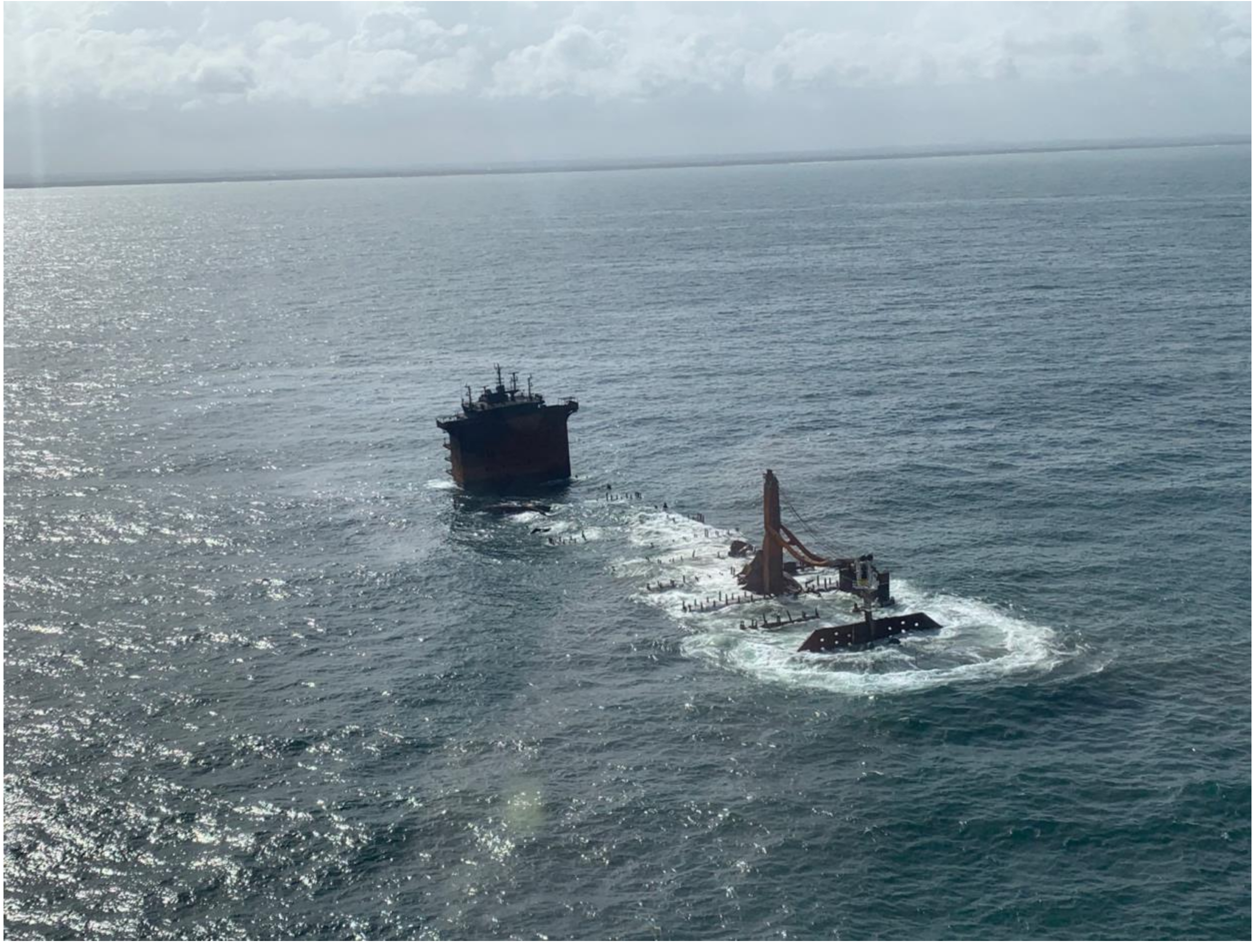

Figure S1. *M/V X-Press Pearl* after sinking photographed on July 10, 2021 (photo credit: Conor Bolas, ITOPF).

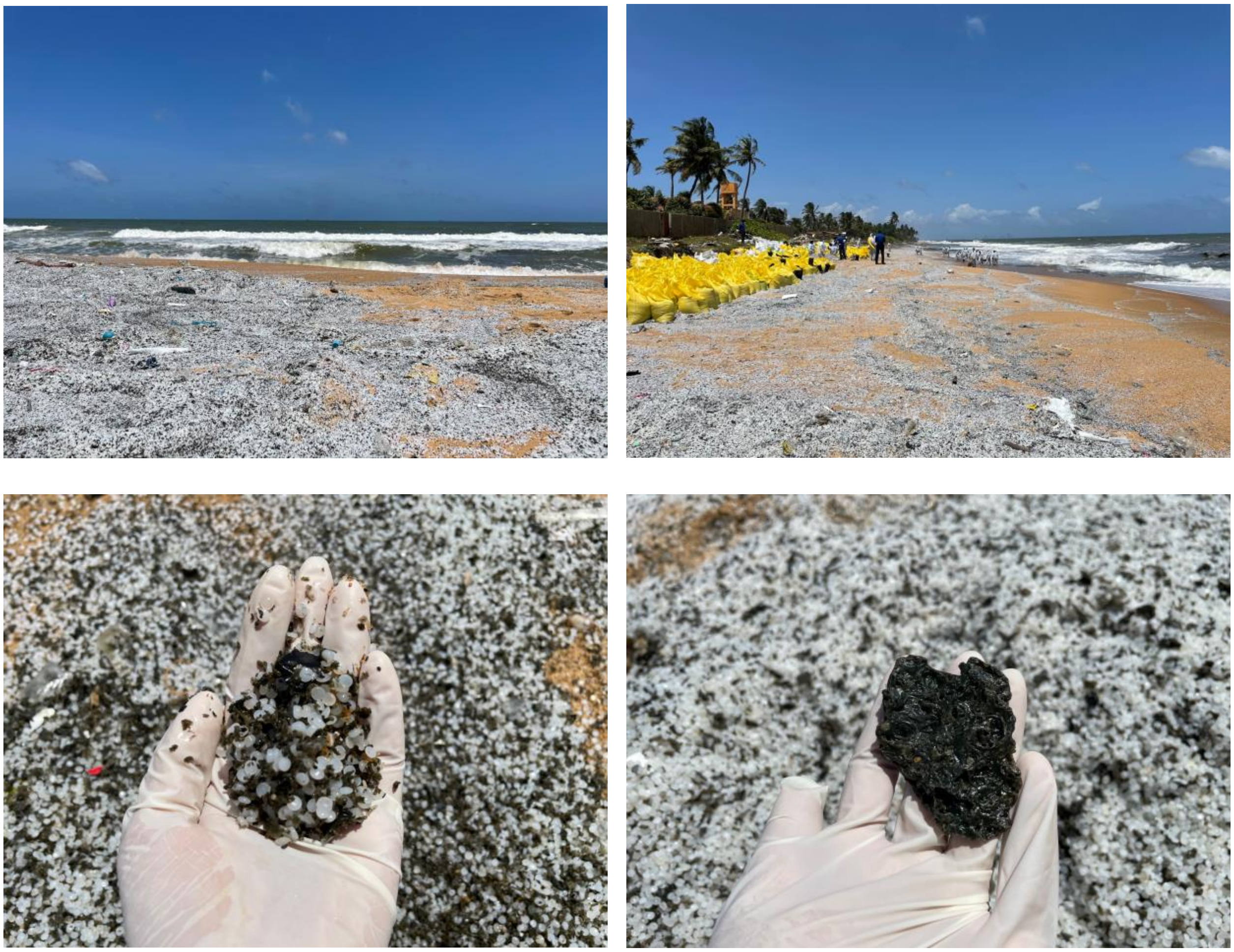

Figure S2. Additional images of the nurdle spill and samples from Pamunugama Beach photographed on May 25, 2021. Photographs show that the burnt plastic can be much larger or smaller than the unburnt nurdles. The small black and brown fragments and flakes are burnt pieces. The plastic shown in the bottom right photograph is an example of a "combustion remnant", a large agglomeration (>10x larger than nurdles) of burnt plastic.

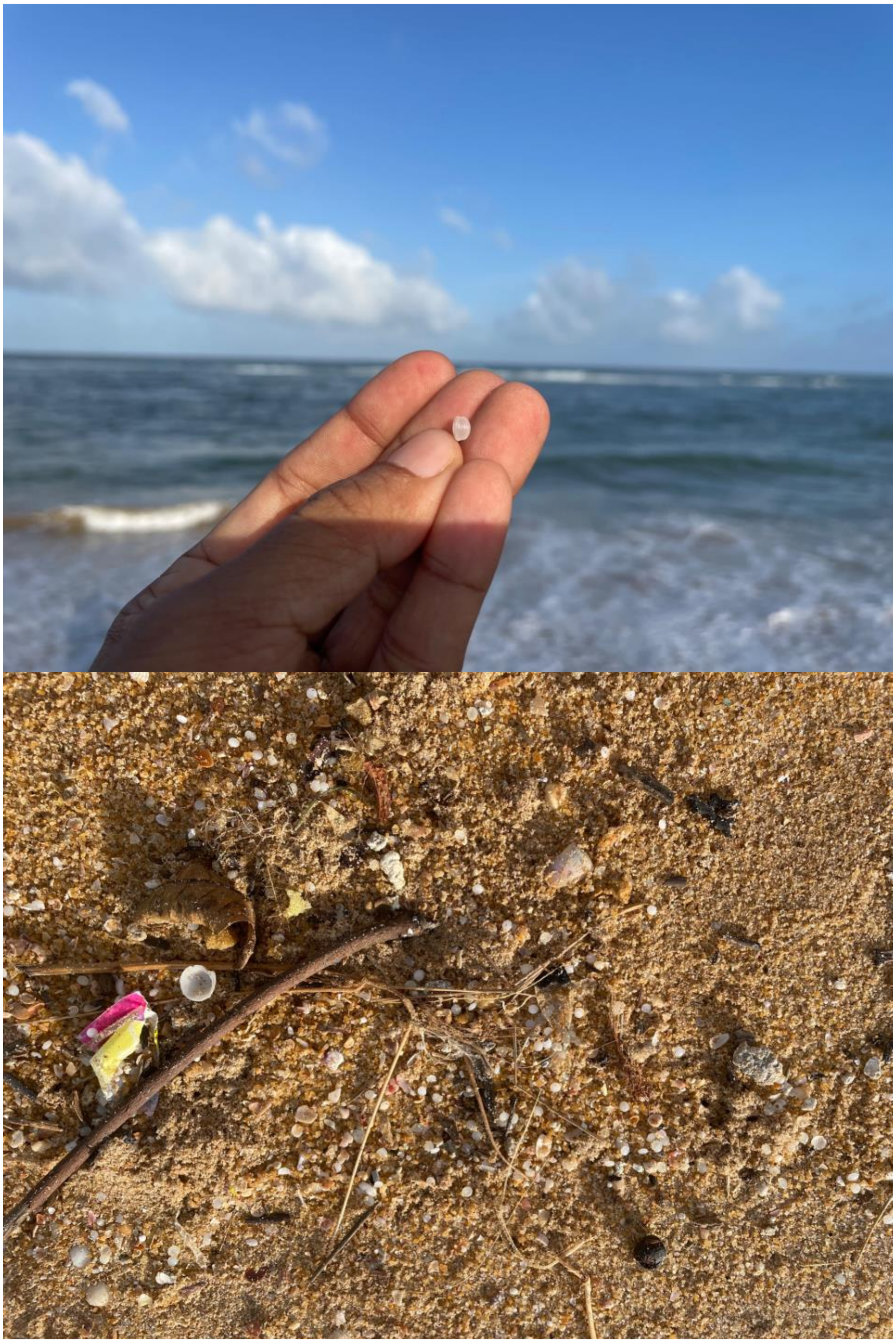

Figure S3. Additional photographs of the nurdle spill and samples from Dehiwela Beach taken on June 23, 2021. Photographs show the size of the unburnt nurdles (top) and their littering of beaches (bottom).

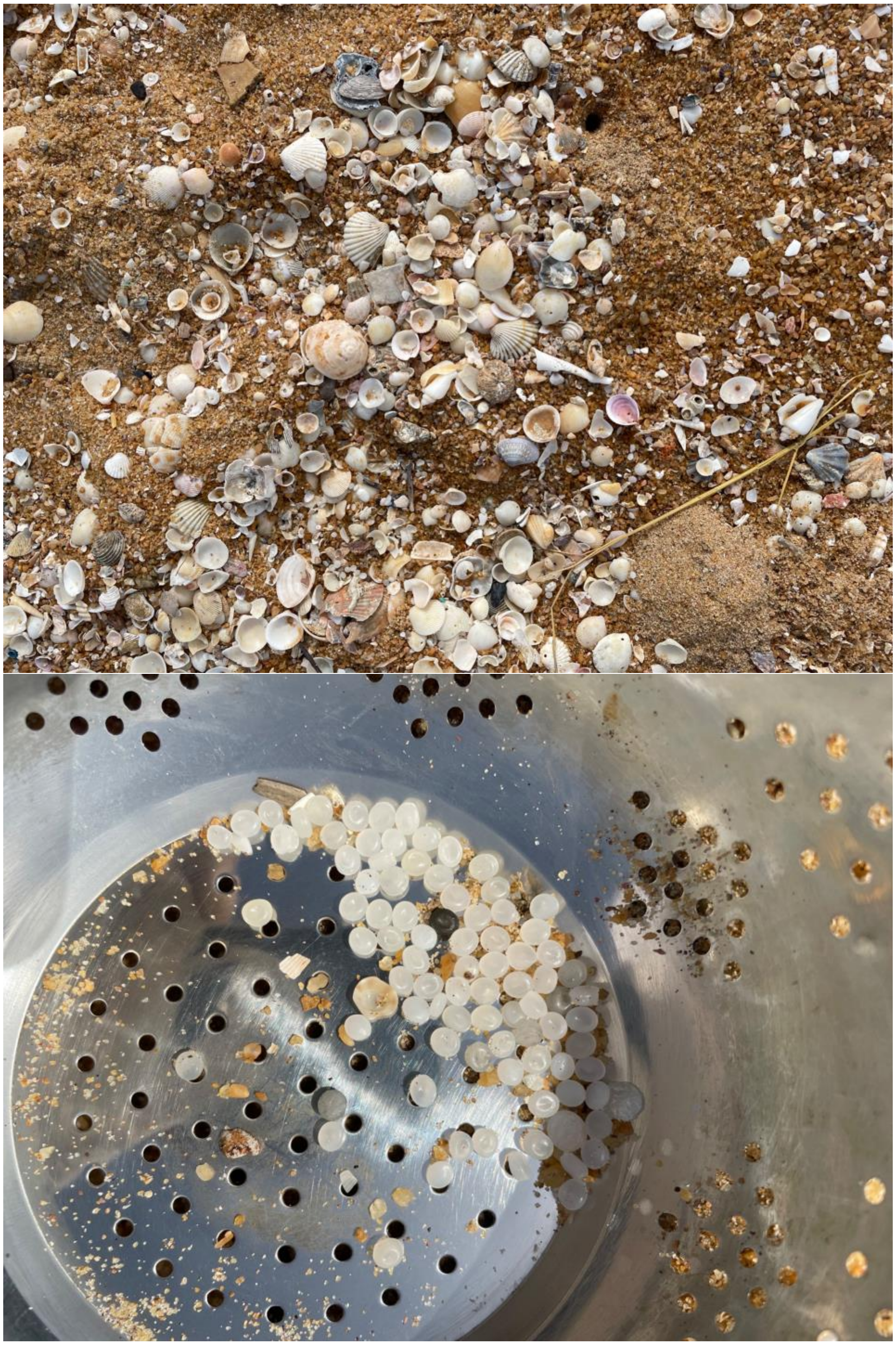

Figure S4. Additional photographs of the nurdle spill from Mt. Lavinia taken on July 3, 2021. Photographs show the unburnt nurdles can be camouflaged among shells (top) and divots can be seen in the unburnt nurdles (bottom).

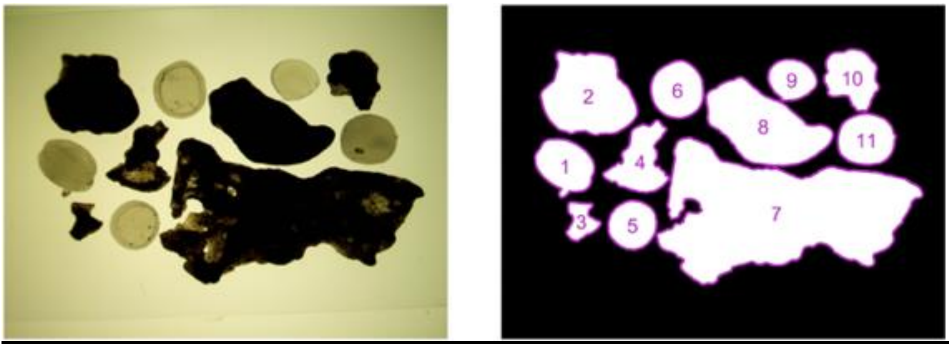

Sample ID	Mass (mg)	Major Axis (mm)	Minor Axis (mm)	Area (mm^2)	Perimeter (mm)
1	36.7	5.1	4.0	15.6	15.2
2	44.8	7.6	6.7	38.7	24.4
3	3.7	3.3	2.5	5.5	10.5
4	8.7	6.2	4.2	16.9	19.6
5	15.3	4.0	3.8	12.1	12.4
6	19.2	4.6	4.3	15.3	14.0
7	106.4	22.2	9.7	145.4	66.8
8	39.3	10.6	5.4	42.9	27.0
9	8.4	3.9	3.3	9.9	11.5
10	17.2	5.0	4.1	15.6	15.7
11	28.2	4.7	4.2	15.4	14.1

Figure S5. Comparison of burnt plastic and unburnt nurdle metrics for shape and size.

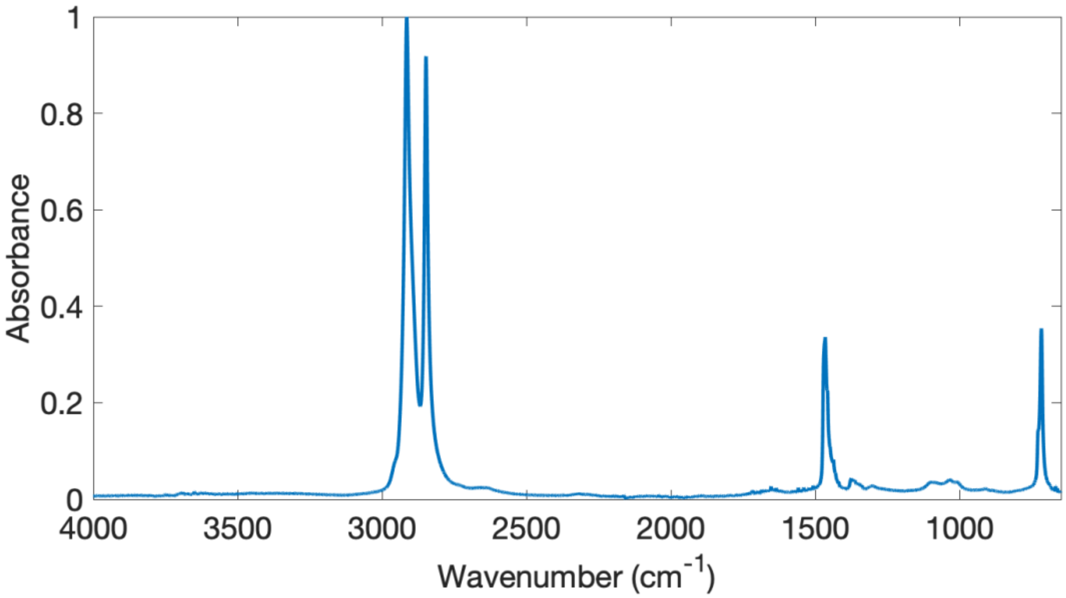

Figure S6. Attenuated total reflection-Fourier transform infrared (ATR-FTIR) spectrum of an unburnt nurdle. This spectrum is consistent with that of polyethylene.[1] Measurements were conducted using an Agilent Technologies Cary 630 FTIR spectrometer coupled to a D-ATR diamond crystal accessory with a single reflection sensor and a sample press. Absorbance spectra were collected using 32 scans at a 2 cm^{-1} resolution measuring between 650 and 4000 cm^{-1}.

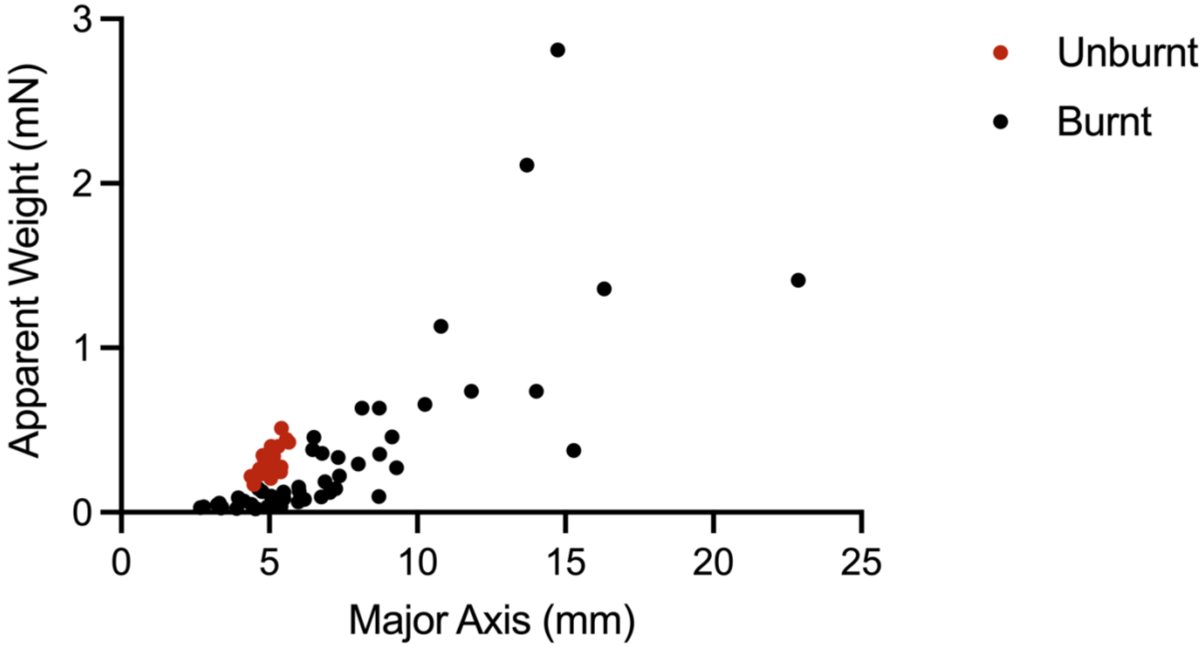

Figure S7. Estimated apparent weight of nurdle samples shown in **Figure 2B** plotted against major axis length. Apparent weight is the weight of the nurdles subtracted from the buoyant force of the nurdles assuming they are submerged in seawater. Positive values refer to positively buoyant nurdles; larger apparent weight values correspond to more buoyant nurdles.

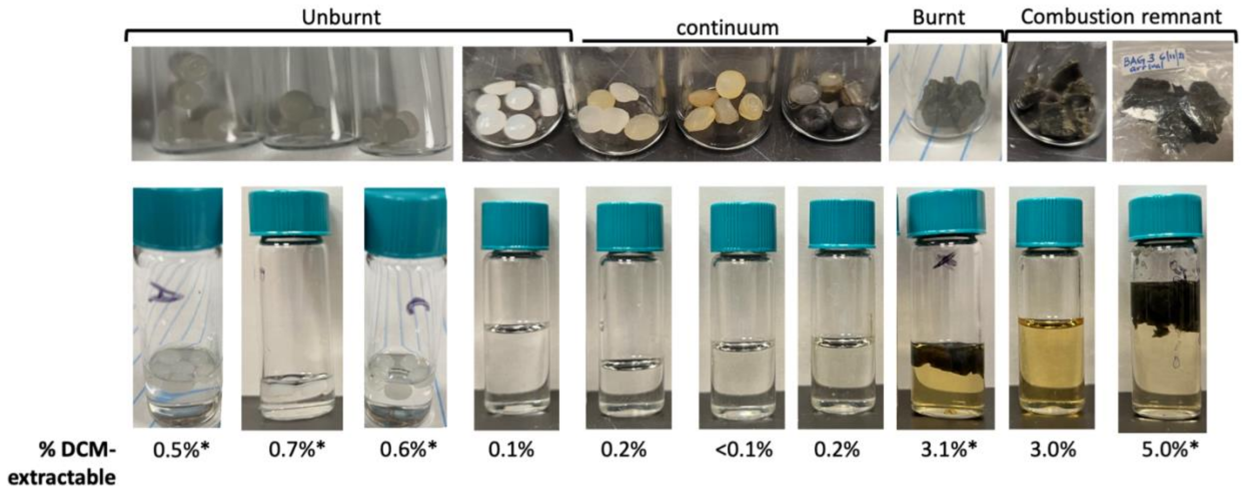

Figure S8. Images of solvent extracts. The extracts for the visibly unburnt nurdles were clear, for the visibly burnt plastic were a faint yellow, and for the extracts from pieces of the combustion remnants were a bolder yellow. The combustion remnants were pieces of excised material from large agglomerations (>10x larger than nurdles) of burnt plastic like that shown in **Figure S2**. All samples were solvent-extracted with dichloromethane (DCM). Asterisks (*) indicate extracts that were used for GC×GC analysis shown in **Figure 3**.

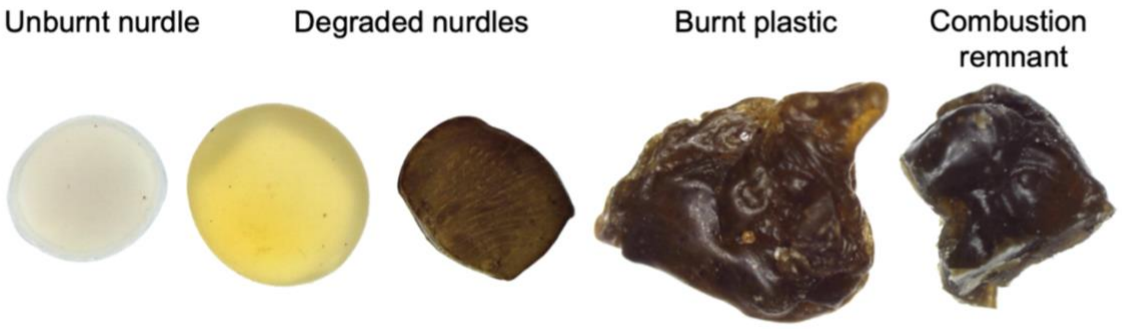

Elemental Analysis (%)

**%C**	85.2	84.9	84.8	82.2	81.1
**%H**	13.8	14.7	14.5	14.2	14.5
**%N**	1.1	0.5	0.7	0.5	0.4
**%Other**	0.0	0.0	0.0	3.1	4.0
**H/C**	1.94	2.08	2.05	2.07	2.14

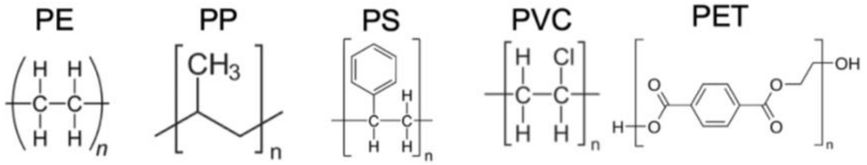

Theoretical

**%C**	85.6	85.6	92.2	38.4	62.5
**%H**	14.3	14.3	7.7	4.84	4.2
**%N**	0.0	0.0	0.0	0.0	0.0
**%Other**	0.0	0.0	0.0	56.7	33.3
**H/C**	2.01	2.01	1.00	1.51	0.81

Figure S9. Images of samples used for elemental analysis of carbon, hydrogen, and nitrogen and their associated percent carbon (%C), percent hydrogen (%H), percent nitrogen (%N), and percent other material (%Other), and H/C ratio. All samples included a fraction (≤~1%) of nitrogen and should be explored further. The theoretical elemental compositions and H/C ratio for polyethylene (PE), polypropylene (PP), polystyrene (PS), polyvinylchloride (PVC), and polyethylene terephthalate (PET) are provided for reference. Elemental analysis measurements were performed by Midwest Microlabs (Indianapolis, IN).

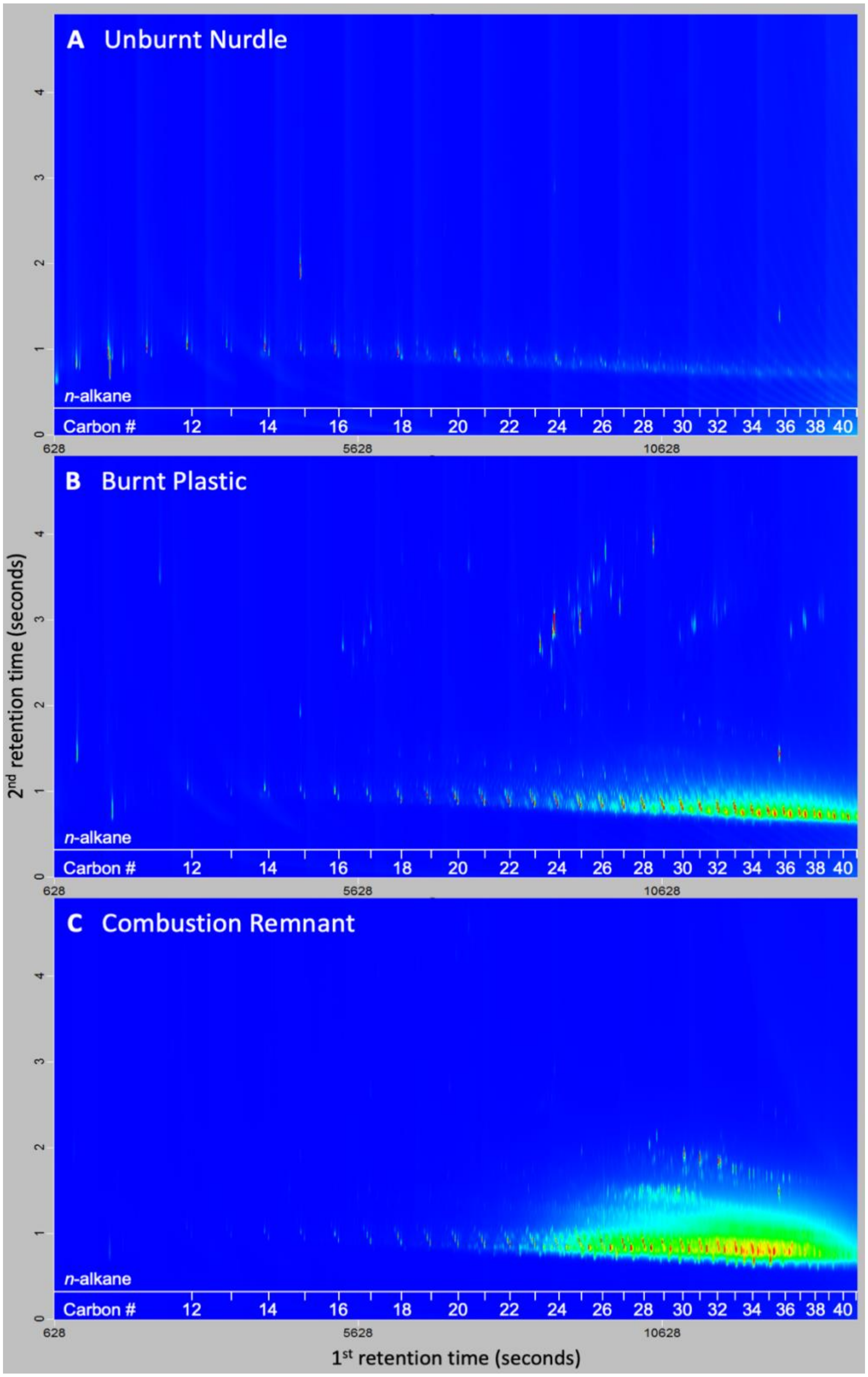

Figure S10. GC×GC-FID chromatograms of the dichloromethane-extractable material from the unburnt nurdles (A), burnt plastic (B), and combustion remnant (C) presented in **Figure 3**.

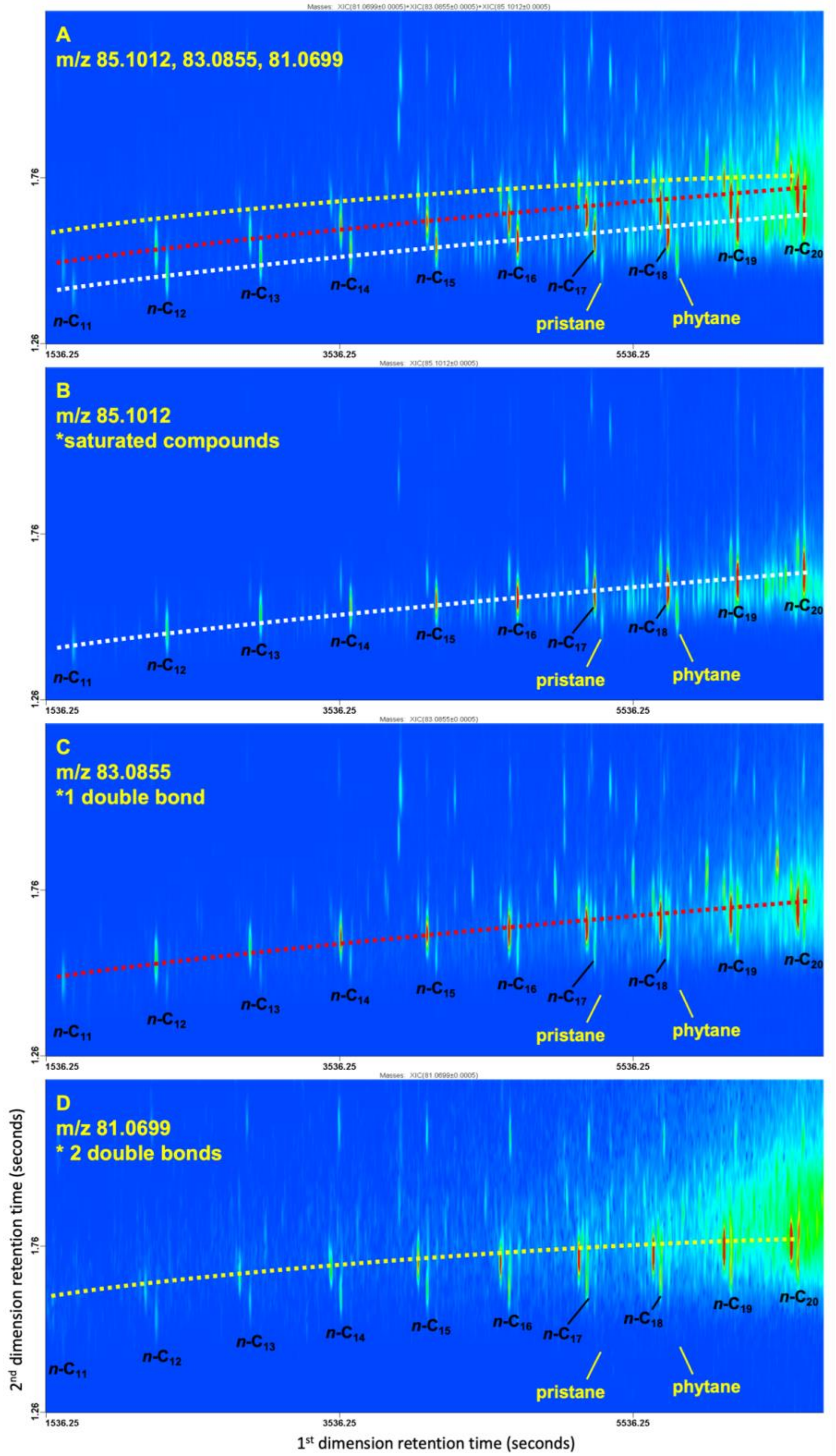

Figure S11. GC×GC-HRT chromatograms of hydrocarbon "fairways" (A) highlighting fairways of *n*-alkanes (B), alkenes (C), and alkadienes (D) in the combustion remnant presented in **Figure 3**. The chromatograms presented here are consistent with those for the burnt plastic that included these fairways, too.

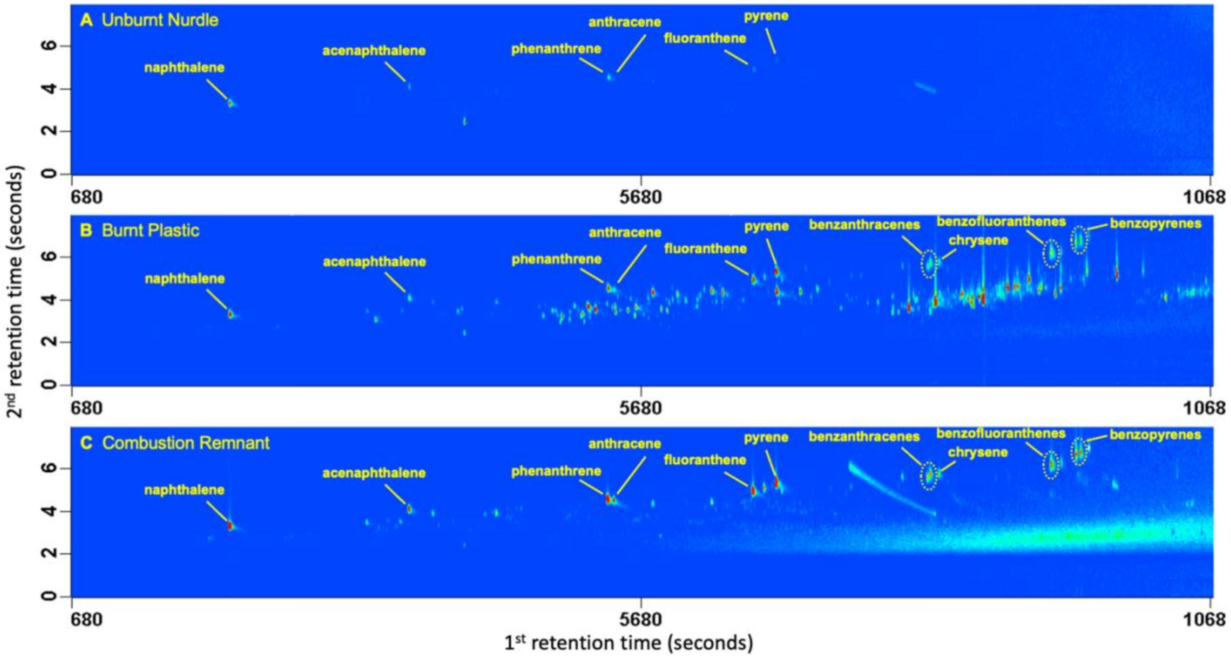

Figure S12. GC×GC-HRT chromatograms highlighting polycyclic aromatic hydrocarbons (PAHs) in the dichloromethane-extractable material from the unburnt nurdles (A), burnt plastic (B), and combustion remnant (C) presented in **Figure 3**.

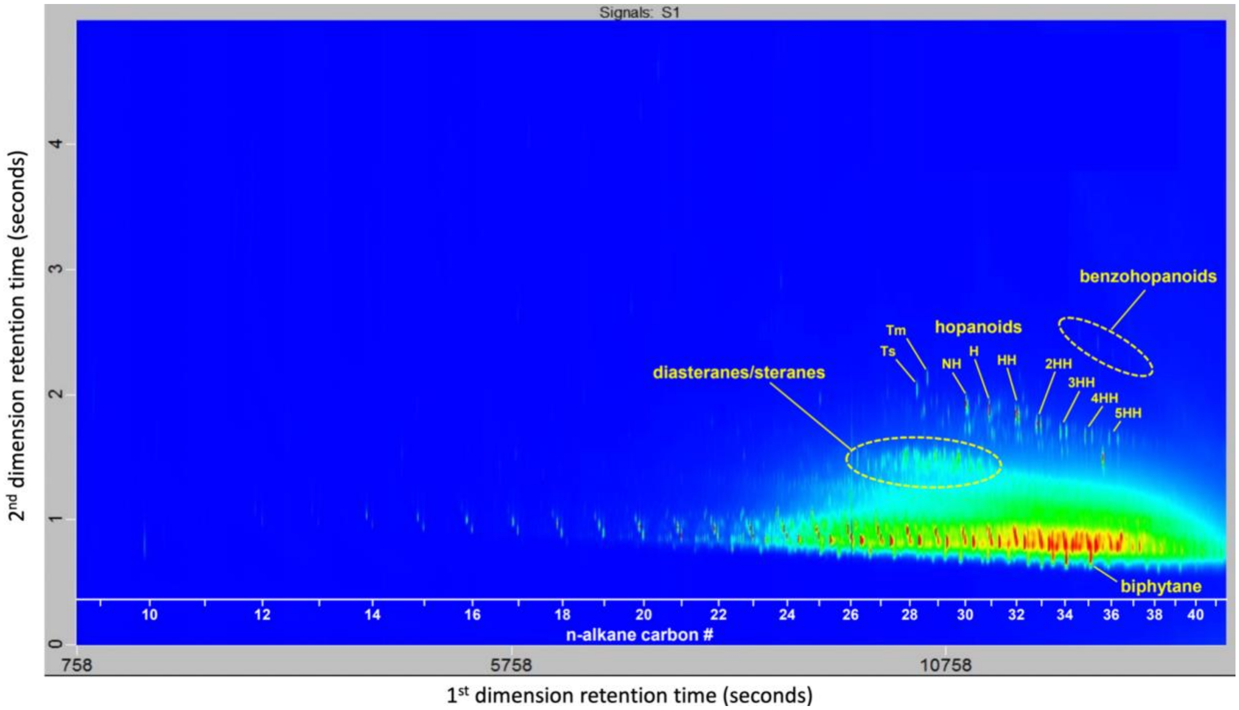

Figure S13. GC×GC-FID chromatogram highlighting petroleum-derived biomarkers identified in the dichloromethane-extractable material from the combustion remnant presented in **Figure 3**.

1. Materials and Methods

1.1 Plastic Samples

Plastic samples that washed ashore from the *M/V X-Press Pearl* cargo ship fire were collected from Pamunugama Beach, Sri Lanka on May 25, 2021. Plastic samples were sorted into two groups, "unburnt" and "burnt" based qualitatively on color. Unburnt nurdles appeared as a mixture of both clear and translucent, and white and opaque plastics in similar condition to new resin pellets. Burnt plastic appeared as dark, irregularly shaped pieces, both smaller and larger in size than the unburnt nurdles.

1.2 Morphometric Analysis

Image analysis was used to characterize the morphology of the plastic samples. Fifty samples of each plastic group (unburnt nurdles and burnt plastic) were selected to cover a range of shapes, sizes, colors and clarities. To remove any excess moisture, samples were dried in a fume hood for 24 hours before image analysis. Samples were imaged in sets of six using an AmScope SZM Series Stereo Microscope with an AMScope C-Mount Reduction Lens camera attachment. During imaging, no specific orientation of the sample was used. The morphometrics for each sample were measured in MATLAB. To detect individual samples in each image, the image was first converted to binary using an empirically set threshold. Then the MATLAB function "regionprops" was used to measure physical parameters of each sample including, major axis length, minor axis length, circularity, projected area, maximum Feret diameter, minimum Feret diameter, aspect ratio, effective spherical diameter, equivalent diameter from area, and projected perimeter. To confirm morphometrics determined by image analysis, a digital caliper was used to manually verify both axis lengths and Feret diameters.

1.3 Density Analysis

Density determination was modified from Morét-Ferguson et al.[2] Samples were placed in containers of distilled water and methanol was added dropwise until the samples were neutrally buoyant. Then aliquots of known volume of the water-methanol solution were massed on an analytical balance. The density of the neutrally buoyant sample was thus equal to the ratio of the mass of the water-methanol solution over the volume of that mass of solution. While density can be used to distinguish between different types of plastics, exposure to fire and other factors related to the spill may affect this approach.

1.4 Calculation of Buoyancy

The buoyant force on each plastic sample was estimated, assuming it was submerged in seawater, where the force from buoyancy is $F_B=\rho V g$, where ρ is the density of seawater (approximately 1025 kg/m^3), V is the volume of the plastic particle, and $g = 9.81$ m/s^2 is the acceleration due to gravity. The measured mass (m) of each sample was converted to volume with the relationship $V = \frac{m}{\rho_p}$, where ρ_p is the estimated density of the plastic (either low density of 930 kg/m^3 or high density of 960 kg/m^3). Because ρ and g are constant, the buoyancy is directly proportional to the volume of the plastic particles, and therefore the largest particles are the most buoyant. Buoyancy acts against the weight of the object, and therefore the net vertical force on a static, submerged plastic particle is the apparent weight (AW), where $AW = mg - \rho g V$, which is plotted in **Figure S7**.

1.5 Solvent Extractable Material

Plastic samples from along the burnt nurdle continuum as defined by sample color were evaluated to assess whether visual appearance translated to differences in solvent-extractable material. 5 pieces of each sample type (unburnt nurdles, degraded nurdles, burnt plastic, and combustion remnants) were incubated with dichloromethane overnight. The total mass of each group was kept constant and quadruplicate extractions were made of unburnt nurdles. Duplicate extractions were made of all other groups (**Figure S8**).

1.6 Loss on Ignition Analysis

Loss on ignition (LOI) measurements were used to determine the inorganic content of the visibly unburnt nurdles and the combustion remnants, with the assumption that any remaining ash represented inorganic materials (e.g., inorganic additives, sand, etc.). This procedure was based on ASTM D 2974-87, the method for determining the ash content of organic matter[3], modified for plastics, as described in Walsh et al.[4] Briefly, ~500 mg of each sample type (n=3) was cleaned in MilliQ water to remove any sand and debris, then placed in a pre-combusted, pre-weighed ceramic crucible and combusted at 450 °C for 4 hours. The ash-containing crucible was then re-weighed to determine the amount of ash remaining.

1.7 Comprehensive Gas Chromatography (GC×GC) Analysis

GC×GC-FID Method

GC×GC-FID chromatographic analyses were performed on a Leco instrument system consisting of an Agilent 7890A GC configured with a split/splitless auto-injector (7683B series) and a dual stage cryogenic modulator (Leco, Saint Joseph, Michigan). Samples were injected in splitless mode. The cold jet gas was dry N_2 chilled with liquid N_2. The hot jet temperature offset was 5 °C above the temperature of the main GC oven and the inlet temperature was isothermal at 310 °C. Two capillary GC columns were utilized in this GC×GC experiment. The first-dimension column was a Restek Rxi-1ms, (60-m length, 0.25 mm I.D., 0.25 μm df) and second-dimension separations were performed on a 50% phenyl polysilphenylene-siloxane column (SGE BPX50, 1.2-m length, 0.10 mm I.D., 0.1 μm df). The temperature program of the main oven was held isothermal at 65 °C (12.5 min) and was then ramped from 65 to 340 °C at 1.25 °C min^{-1}. The second-dimension oven was isothermal at 70 °C (12.5 min) and then ramped from 70 to 345 °C at 1.25 °C. The hot jet pulse width was 1.0 seconds, the modulation period was 6.5 seconds with a 2.25 second cooling period between stages, GC×GC-FID data was sampled at an acquisition rate of 100 data points per second. The carrier gas was hydrogen (H_2) at a flow rate of 1 mL min^{-1}.

GC×GC-HRT Method

GC×GC-HRT chromatographic analysis was performed on a Leco Pegasus GC×GC-HRT 4D system consisting of an Agilent 7890B GC configured with a Leco LPAL3 split/splitless auto-injector system and a dual stage cryogenic modulator (Leco, Saint Joseph, Michigan). Samples were injected in splitless mode. The cold jet gas was dry N_2 chilled with liquid N_2. The hot jet temperature offset was 25 °C above the temperature of the main GC oven and the inlet temperature was isothermal at 310 °C. Two capillary GC columns were utilized in this GC×GC experiment. The first-dimension column was a Restek Rxi-1ms, (60-m length, 0.25 mm I.D., 0.25 μm df) and second-dimension separations were performed on a 50% phenyl polysilphenylene-siloxane column (SGE BPX50, 1.2-m length, 0.10 mm I.D., 0.1 μm df). The temperature program of the main oven was held isothermal at 75 °C (12.5 min) and was then ramped from 75 to 315 °C at 1.25

°C min^{-1}. The second-dimension oven was isothermal at 88 °C (12.5 min) and then ramped from 88 to 328 °C at 1.25 °C. The hot jet pulse width was 2.4 seconds, the modulation period was 8.00 seconds with a 1.6 second cooling period between stages, GC×GC-HRT data was sampled at an acquisition rate of 194.44 spectra per second in the mass range of 40 to 500 atomic mass units (amu). The carrier gas was helium (He) at a flow rate of 1 mL min-1. HR-TOF data was sampled at an acquisition rate of 200 spectra per second (actual data collection rate was 194.44 spectra per second) in the mass range of 40 to 500 amu. The ionization method was EI with an Electron Energy of -70 Volts and the Extraction Frequency was 1.75 kHz.

GC×GC QA/QC

We used National Institute of Standards and Technology (NIST) standard reference materials SRM-1582 (Petroleum Crude Oil) to calibrate and validate our GC×GC instruments. GC×GC performance was monitored on all instruments using SRM-1582. We routinely intersperse SRM-1582 samples with analytical samples and monitor a suite of biomarker ratios in order to confirm that the instruments are stable and operating as expected.

GC×GC-HRT Calibration

GC×GC-HRT mass spectra were calibrated using a continuous flow of perfluorotributylamine (PFTBA) introduced by opening a valve into the electron ionization (EI) source in the GC×GC-HRT instrument. GC×GC-HRT data collected throughout each EI run is calibrated with respect to the molecular ion (+1 charge state) of eight perfluorinated compounds (CF_3, C_2F_4, C_2F_5, C_3F_5, C_4F_9, $C_5F_{10}N$, $C_8F_{16}N$, and $C_9F_{20}N$). The mass values for singly charged ions in the mass range of 40-650 amu, with a relative abundance at least ten times the signal to noise ratio of the baseplane were acquired and stored. Additional GC×GC-HRT data processing was performed using a petroleomics mass spectral data analysis application for LECO's ChromaTOF software.

References

(1) Jung, M. R.; Horgen, F. D.; Orski, S. V.; Rodriguez C., V.; Beers, K. L.; Balazs, G. H.; Jones, T. T.; Work, T. M.; Brignac, K. C.; Royer, S.-J.; et al. Validation of ATR FT-IR to Identify Polymers of Plastic Marine Debris, Including Those Ingested by Marine Organisms. *Mar. Pollut. Bull.* **2018**, *127*, 704–716. https://doi.org/10.1016/j.marpolbul.2017.12.061.

(2) Morét-Ferguson, S.; Law, K. L.; Proskurowski, G.; Murphy, E. K.; Peacock, E. E.; Reddy, C. M. The Size, Mass, and Composition of Plastic Debris in the Western North Atlantic Ocean. *Mar. Pollut. Bull.* **2010**, *60* (10), 1873–1878. https://doi.org/10.1016/j.marpolbul.2010.07.020.

(3) ASTM International. ASTM D2974 - 14 - Standard Test Methods for Moisture, Ash, and Organic Matter of Peat and Other Organic Soils. West Conshoshocken, PA 2014. https://doi.org/10.1520/D2974-14.

(4) Walsh, A. N.; Reddy, C. M.; Niles, S. F.; McKenna, A. M.; Hansel, C. M.; Ward, C. P. Plastic Formulation Is an Emerging Control of Its Photochemical Fate in the Ocean. *Environ. Sci. Technol.* **2021**, *55* (18), 12383–12392. https://doi.org/10.1021/acs.est.1c02272.
